# Challenging diagnosis of cervical vagal nerve schwannoma

**DOI:** 10.1093/jscr/rjac084

**Published:** 2022-04-03

**Authors:** Lina Pankratjevaite, Niloofar Sherazi Dreyer, Albertas Dauksa, Valdas Sarauskas

**Affiliations:** Department of Breast Surgery, Rigshospitalet, Copenhagen University Hospital, Copenhagen, Denmark; Department of Breast Surgery, Herlev and Gentofte Hospital, Copenhagen University Hospital, Herlev, Gentofte, Denmark; Department of Otorhinolaryngology, Head and Neck Surgery, Rigshospitalet, Copenhagen University Hospital, Copenhagen, Denmark; Department of Surgery, Medical Academy, Hospital of Lithuanian University of Health Sciences, Kaunas, Lithuania; Department of Pathology, Medical Academy, Hospital of Lithuanian University of Health Sciences, Kaunas, Lithuania

## Abstract

Schwannoma arising from vagal nerve is a rare tumour. It is a slow-growing, benign mass, but rarely it might undergo malignant transformation. We report a case of a 55-year-old woman with asymptomatic Xth cranial nerve schwannoma in the left side of the neck. Initially, during the ultrasound examination, the tumour was misconceived to be a malignant lymph node. The patient underwent complete surgical excision of it. Histopathological examination revealed typical features of schwannoma. Clinical diagnose of cervical vagal nerve schwannoma is difficult. Magnetic resonance imaging is as an accurate diagnostic tool for these tumours. Surgical excision is the treatment of choice.

## INTRODUCTION

Schwannoma (syn. neurilemoma, neuroschwannoma) is a benign, encapsulated neoplasm originating from Schwann cells of peripheral, cranial or autonomic nerves [1]. Parapharyngeal space is the most common place of the head and neck region where extracranial schwannomas arise [2]. Vagal nerve (Xth cranial nerve) schwannoma is a rare, slow-growing tumour [1, 3, 4]. However, rarely it might become malignant [4]. Vagal nerve schwannoma most often affects people between the third and fifth decades [1, 5, 6]. There is no sex-related predisposition [1, 5, 6]. It is usually asymptomatic [6] but sometimes hoarseness, pain, or cough may be present [4]. In advanced cases, symptoms, such as dysphonia, dysphagia, dyspnoea, pharyngeal or airway obstruction, may develop and are suggested to be correlated with schwannoma size. Magnetic resonance imaging (MRI) is a gold standard used to assess vagal nerve schwannomas and to evaluate their extent [1, 3, 6]. Surgical excision is the treatment of choice for these tumours [3, 6].

## CASE REPORT

A 55-year-old woman was admitted to the ambulatory for elective ultrasound (US) to assess the thyroid gland and neck. The patient complained of a palpable lump in the left side of the neck. Her past medical history was significant for thyroid nodules diagnosed 4 years ago, arterial hypertension and vertebrobasilar insufficiency lasting few years. There was no family history of malignancy. Her social history was non-significant.

**Figure 1:**
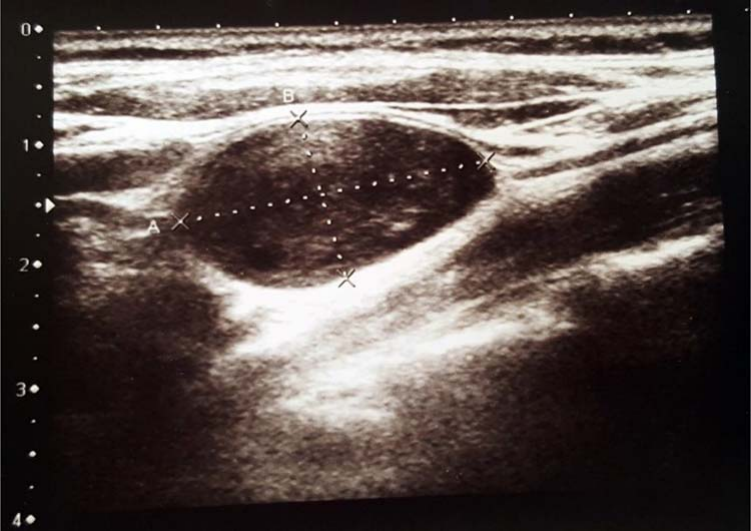
US showed ovoid-shaped mass.

US revealed normal size, with hypoechogenic nodules thyroid gland and a heterogenic 2.6 × 1.4-cm size ovoid shape mass in the left side of the neck (Fig. 1). The tumour was misunderstood to be a lymph node and malignant process was suspected. Fine needle aspiration cytology (FNAC) of the mass was performed, but it was inconclusive. The patient was referred to the Department of Surgery for suspected malignant lymph node excision. During physical examination, her blood pressure was 150/90 mmHg and heart rate was 88 times per min. Her electrocardiography showed sinus rhythm. Blood laboratory findings were within normal limits. Clinical examination revealed firm, non-tender, ~3 × 2-cm size mass in the left side of the neck.

The patient underwent surgery. Under general anaesthesia, a cervical incision was done. An encapsulated, well-circumscribed, yellowish, ovoid-shaped, 3 × 1.5-cm size mass was found between the internal jugular vein and the carotid artery. It originated from the vagal nerve. During the excision of the mass, the patient’s pulse decreased from 90 beats per min to 20 beats per min. After the mass was removed, the patient’s pulse got normalized. The tumour was removed with a nerve-sparing technique.

**Figure 2:**
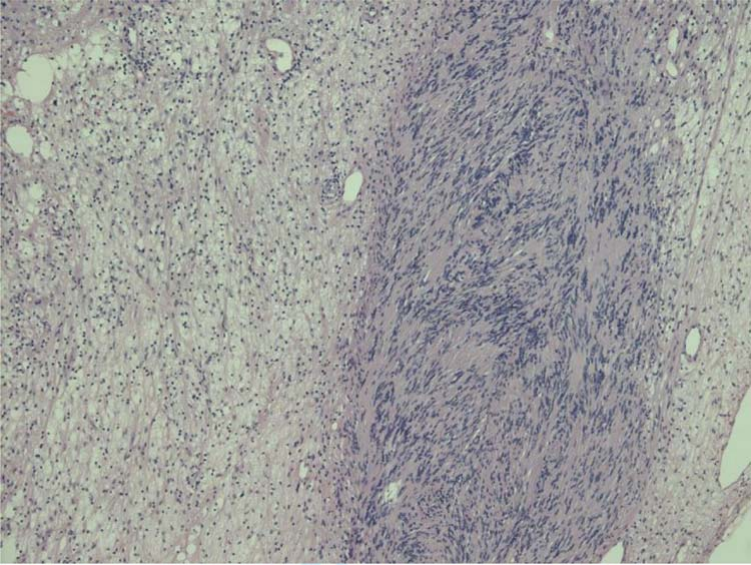
Biphasic tumour: compact hypercellular Antoni A area (right) and myxoid hypocellular Antoni B area (left).

**Figure 3:**
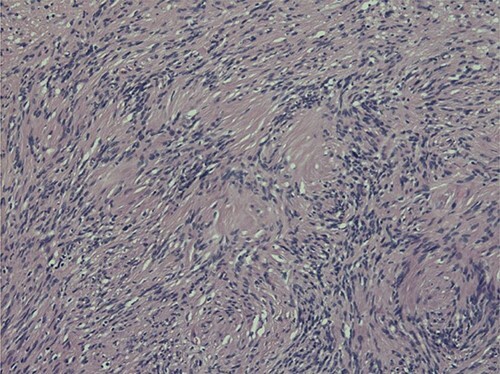
Nuclear palisading around fibrillary process (Verocay bodies) is seen in cellular area; cells are narrow, elongated and wavy with tapered ends interspersed with collagen fibres.

Histopathological examination revealed typical features of schwannoma (Figs 2 and 3). Tumour was well demarcated, encapsulated, composed of spindle cells, organized in a palisading fashion and had hypocellular myxoid component with large vessels. Tumour cells had an ill-defined cytoplasm and elongated nucleus. There was no mitotic activity.

Post-operatively, the patient complained of coughing which disappeared after 2 months. At follow-up, 2 years after the surgery, there was no recurrence of the tumour.

## DISCUSSION

Schwannoma is a benign tumour originating from cranial, peripheral or autonomic nerves except optic and olfactory nerves [1, 7]. Usually, it is solitary but sometimes it might be multiple [5, 7]. About 25% of the cases of schwannoma occur in the head and neck area [8]. Vagal nerve schwannoma is a rare tumour [1, 3, 4, 6–9], which rarely undergoes malignant transformation [4]. It most often affects people between the third and fifth decades [1, 5, 6]. Both sexes are affected equally [5, 6]. Vagal nerve schwannoma usually is an asymptomatic, slow-growing tumour [1, 4]. Sometimes symptoms, such as hoarseness or paradoxical coughing during palpation of the mass, may be present [1, 4, 6, 7, 10]. This sign is unique to vagal nerve schwannoma [1, 6, 10]. None of these symptoms were present in our case. The patient only complained about asymptomatic neck lump. Some authors say that FNAC should be used as a routine procedure for diagnosing the origin of all neck masses [3]. However, the usefulness of preoperative diagnosis of vagal nerve schwannoma by FNAC is controversial [1, 7, 8]. The quality of the specimen and the experience of cytopathologist influence the preoperative diagnostic accuracy of FNAC [1]. Moreover, sometimes FNAC can be dangerous and if the cervical mass clinically and radiologically is suspected to be benign, it is not recommended to do needle or open biopsy because the treatment is still surgical [8].

Radiologic imaging plays an important role in diagnosing vagal nerve schwannoma. On US images, it appears as a round or elliptical cross-section with a clear border tumour [1]. Computed tomography (CT) shows vagal nerve schwannoma as a well-covered, well-defined mass [1, 4], which is usually of higher attenuation than muscle on contrast-enhanced images [4].

However, MRI is a gold standard to assess vagal nerve schwannomas and to evaluate their extent [1, 3, 6]. MRI evaluation typically shows well-circumscribed mass lying between the internal jugular vein and the carotid artery [6]: isotense or hypointense signal on T1-weighted images and hyperintense signal on T2-weighted images are seen [12]. Moreover, MRI is important for differential diagnosis and treatment planning. It is important to notice, that our patient did not have nervus vagus schwannoma’s symptoms and complained only of a palpable lump in a neck. A US was performed and the mass in the neck was misunderstood to be a malignant lymph node. Due to inconclusive FNAC, the patient was sent directly to surgical lymph node biopsy and during the surgery it was discovered that it was not a lymph node. Our case shows that preoperative MRI or CT scan of the neck can be very helpful for diagnosis and for planning the treatment.

Macroscopically schwannoma looks like yellowish-white, well-circumscribed mass [1, 6]. Microscopically, the baseline features of schwannoma are Antoni type A tissue and Antoni type B tissue [1, 4]. Necrosis, haemorrhage and cystic degeneration are other specific features [1, 4].

Anamnesis, imaging studies play an important role in the differential diagnosis of vagal nerve schwannoma. The differential diagnosis of vagal nerve schwannoma includes neurofibromas, metastatic lymph nodes, lymphoma, paragangliomas, glomus vagale tumours/carotid body tumours, schwannomas of cervical sympathetic origin, branchial cleft anomalies, cysts of the structures of the neck (e.g. thyroid and parathyroid) and vascular malformations of the neck [4]. Blood samples and sometimes even genetic assessment may be necessary for diagnosis. It is very important to distinguish vagal nerve schwannoma from other diseases as it can influence further examination and the treatment. For example, if on radiologic imaging schwannoma could not be distinguished from neck paraganglioma, FNAC is not recommended and it is even dangerous to be performed due to the risk of hypertensive crisis after FNAC. Moreover, it is important to differentiate schwannoma from hyperfunctional neck paraganglioma, as the latter may be associated with intraoperative hemodynamic instability and hypertensive crisis due to acute release of catecholamines during surgery.

The treatment of choice of vagal nerve schwannoma is a complete surgical excision [1, 3, 4, 8]. However, regular observation with imaging could be offered to poor surgical candidates and asymptomatic, older patients.

Nerve sparing dissection should be performed for benign schwannoma [1, 6, 8]. ‘Subtotal resection’ with or without intracapsular enucleation may be performed [13]. This technique allows higher rate of nerve preservation and lower rates of post-operative vocal fold palsy [14]. If it is not possible to spare the nerve, end-to-end anastomosis or interposition of nerve graft might be done [15]. For malignant schwannomas, the best treatment option is wide excision where possible [15]. Moreover, vagal nerve schwannoma might be removed by an endoscopic gasless unilateral axillo-breast approach [16]. However, excision of vagal nerve schwannoma has a risk of vocal cord paralysis [6, 15]. The intraoperative neurostimulation techniques as intermittent intraoperative nerve monitoring and continuous monitoring may reduce the risk of injury to the nerves and prevent from post-operative neurological complications, such as true vocal cord paralysis/palsy, hoarseness and dysphagia [8, 17]. In present case, vagal nerve schwannoma was completely excised; however, the patient post-operatively complained of cough that spontaneously disappeared within 2 months.

Recurrence risk following vagal schwannoma surgery is extremely low and there are no data that present recurrence following the specific choice of surgery [14].

## CONCLUSIONS

Vagal nerve schwannoma is a benign, usually asymptomatic tumour. Rarely, it might become malignant. MRI is a gold standard to assess preoperative diagnosis of cervical vagal schwannoma. FNAC should be performed with precaution. Complete excision of the tumour is the treatment of choice. We recommend to perform surgery with intraoperative nerve monitoring.
